# LTT and HLA testing as diagnostic tools in Spanish vancomycin-induced DRESS cases: A case-control study

**DOI:** 10.3389/fphar.2022.959321

**Published:** 2022-10-20

**Authors:** Teresa Bellón, Victoria Lerma, Javier Guijarro, Elena Ramírez, Celia Martínez, Carmelo Escudero, Ana M. Fiandor, Ruth Barranco, Manuel de Barrio, Francisco de Abajo, Rosario Cabañas, PIELenRed Study Group

**Affiliations:** ^1^ Drug Hypersensitivity Laboratory, Institute for Health Research Hospital Universitario La Paz (IdiPaz), Madrid, Spain; ^2^ Clinical Pharmacology Unit, Hospital Universitario Príncipe de Asturias, Madrid, Spain; ^3^ Department of Biomedical Sciences, University of Alcalá (IRYCIS), Madrid, Spain; ^4^ Clinical Pharmacology Department, Hospital Universitario La Paz-Carlos III-Cantoblanco, IdiPAZ, School of Medicine, Autonomous University of Madrid, Madrid, Spain; ^5^ Allergy Department, Hospital Universitario Niño Jesús, Madrid, Spain; ^6^ Allergy Department, Hospital Universitario La Paz-Carlos III-Cantoblanco, Madrid, Spain; ^7^ Allergy Department, Hospital Universitario Doce de Octubre, Madrid, Spain; ^8^ Allergy Department, Hospital Gregorio Marañón, Madrid, Spain

**Keywords:** drug hypersensitivity, DRESS, HLA, LTT, severe cutaneous adverse reactions, vancomycin, drug causality algorithm

## Abstract

Drug reaction with eosinophilia and systemic symptoms (DRESS) is a severe T-cell-mediated off-target adverse reaction. DRESS cases caused by vancomycin have often been reported. The HLA-A*32:01 allele has been associated with genetic susceptibility to vancomycin-induced DRESS in US citizens of European descent. We have analyzed the association of the HLA-A*32:01 allele in 14 Spanish DRESS cases in which vancomycin was suspected as the culprit drug, and the lymphocyte transformation test (LTT) as an *in vitro* assay to evaluate vancomycin sensitization. The results were compared to vancomycin-tolerant control donors. LTT was performed in 12 DRESS cases with PBMCs from resolution samples available and in a group of 12 tolerant donors. ROC curves determined that LTT is a suitable tool to identify patients sensitized to vancomycin (AUC = 0.9646; *p* < 0.0001). When a stimulation index >3 was regarded as a positive result, contingency tables determined 91% sensitivity, 91.67% specificity, 91% positive predictive value, and 91.67% negative predictive value (*p* = 0.0001, Fisher’s exact test). The HLA A*32:01 allele was determined by an allele-specific PCR assay in 14 cases and 25 tolerant controls. Among the DRESS cases, five carriers were identified (35.7%), while it was detected in only one (4%) of the tolerant donors, [odds ratio (OR) = 13.33; 95% CI: 1.364–130.3; *p* = 0.016]. The strength of the association increased when only cases with positive LTT to vancomycin were considered (OR = 24.0; 95% CI: 2.28–252.6; *p* = 4.0 × 10^−3^). Our results confirm the association of the risk allele HLA-A*32:01 with vancomycin-induced DRESS in Spanish cases, and support LTT as a reliable tool to determine vancomycin sensitization.

## Introduction

Adverse drug reactions are a frequent problem in clinical practice. Among them, skin reactions are observed in 2–3% of hospitalized patients, of which only 2–5% are considered severe (Gomes et al., 2019). Severe cutaneous adverse reactions (SCARs) are T-cell-mediated type IV hypersensitivity reactions. Stevens–Johnson syndrome/toxic epidermal necrolysis (SJS/TEN), acute generalized exanthematous pustulosis (AGEP), and drug reaction with eosinophilia and systemic symptoms/drug-induced hypersensitivity syndrome (DRESS/DiHS) are the conditions of serious concern as, albeit rare diseases, they carry significant morbidity and mortality rates.

In particular, DRESS/DiHS has a mortality rate between 2 and 10 percent (Chen et al., 2010; Cacoub et al., 2011; Kardaun et al., 2013). It typically develops 2–8 weeks after the initiation and continuous drug intake and presents with a variety of cutaneous manifestations, hematological abnormalities such as eosinophilia or atypical lymphocytes, adenopathy, fever, and involvement of one or more organs. The liver is the organ most frequently involved, with the kidneys being second. The heart, lung, pancreas, and central nervous system can also be affected in a small proportion of patients. Sequential reactivation of human herpesvirus (HHV) has been described, particularly HHV-6 and cytomegalovirus (CMV), and it is frequently associated with disease severity (Mizukawa et al., 2019; Shiohara and Mizukawa, 2019). Diagnosis can be challenging as not all the symptoms develop simultaneously (Stirton et al., 2022). The score classifications developed by the Japanese Research Committee on Severe Cutaneous Adverse Reaction (JSCAR) (Shiohara et al., 2007; Shiohara and Kano, 2007) and the European Registry of Severe Cutaneous Adverse Reactions (RegiSCAR) group (Kardaun et al., 2007) are currently used for diagnosis. A few biomarkers such as soluble OX40 (Mitsui et al., 2022) and decreased frequencies of plasmacytoid dendritic cells (pDC) (Hsu et al., 2021) have been recently proposed for diagnosis and prognosis of HHV-6 reactivation.

Aromatic anticonvulsants, allopurinol, and sulfonamides such as sulfamethoxazole or sulfasalazine are common culprit drugs in DRESS cases. However, antimicrobials have also been repeatedly incriminated (Kardaun et al., 2013; Cabañas et al., 2014; Cabañas et al., 2018; Lee et al., 2022).

Pharmacogenetic studies have identified some HLA-I alleles as genetic risk factors for well-characterized type IV hypersensitivity reactions in relationship with certain drugs such as abacavir, carbamazepine, and allopurinol in selected populations, and genetic tests are being implemented to avoid their use and prevent severe reactions in patients at risk (Su et al., 2016; Kuruvilla et al., 2022; Wang et al., 2022); HLA-B*57:01 testing has been conducted prior to prescription in HIV patients (the prototypical case) due to its 100% negative predictive value (NPV) and 55% positive predictive value (PPV) to predict abacavir hypersensitivity (Phillips and Mallal, 2009). Nonetheless, there are no specific biomarkers available for most of the drugs inducing SCARs, and active research is being conducted to identify suitable biomarkers for common inducers of severe reactions.

Vancomycin is a glycopeptide antibiotic extensively used to treat infections caused by gram-positive microbes. It has been frequently reported as a causative agent in DRESS cases (Kardaun et al., 2007; Del Pozzo-Magaña et al., 2022; Madigan and Fox, 2019; Lin et al., 2014).

An analysis of HLA genotypes in North American patients of European descent presenting with vancomycin-associated DRESS identified HLA-A*32:01 as a risk allele to develop this condition (Konvinse et al., 2019). Moreover, it was estimated that only 75 patients would need to be tested to prevent one case. These findings are of high interest for other European populations. As with other genetic associations, the findings would need to be replicated in independent cohorts.

Ascertaining a causative drug is mandatory in severe delayed drug hypersensitivity reactions, due to the high morbidity and mortality upon re-exposure to the culprit drug. However, such identification is often a difficult task, in particular when multiple medications are concomitantly used. Moreover, multiple drug sensitization, including sensitization to drugs introduced during the acute reaction, is frequent in DRESS cases (Gex-Collet et al., 2005; Barbaud et al., 2013). Clinical judgment is not always a reliable tool for drug causality assessment in DRESS. Algorithms such as the Naranjo score (Naranjo et al., 1981) have been developed as an alternative approach to determine the causality likelihood of drugs taken by a given patient (Macedo et al., 2006); however, no DRESS-specific algorithm has been developed yet.

The ENDA/EAACI Drug Allergy Interest Group advises that an LTT should be performed before *in vivo* tests in severe reactions with a suspected T-cell mechanism (Mayorga et al., 2016). Recent results from our group showed that LTT presented a sensitivity of 73% and a specificity of 82% in DRESS cases associated with a variety of drugs when the test was performed after recovery, using the algorithm of the Spanish pharmacovigilance system (ALSEFV) as the gold standard to identify the culprit drugs (Cabañas et al., 2018).

The aim of this study is twofold: 1) to evaluate the usefulness of LTT to vancomycin to support drug causality assessment in DRESS cases in whom this was suspected to be the inducing agent; and 2) to estimate, in a Spanish-European population, the association HLA-A*32:01 with the risk of DRESS induced by vancomycin.

## Methods

### Vancomycin-induced DRESS cases and tolerant control subjects

Fourteen patients recorded in the Spanish registry PIEL*enRed* with a diagnosis of DRESS, in whom vancomycin was considered as the suspected inducing drug, and from which biological samples were available, were included in the study. The diagnosis was validated in all of them by an expert committee (blinded to medications) and classified as possible, probable, or definite cases (a score of two or more) using the DRESS scoring system proposed by RegiSCAR (Kardaun et al., 2014).

Drug causality was assessed using the algorithm of the Spanish pharmacovigilance system (ALSEFV) (Capellà and Laporte, 1993; Cabañas et al., 2018), as recommended by the Spanish guidelines for the management of DRESS (Cabañas et al., 2020). Vancomycin was considered to be related to the adverse reaction when it scored ≥4 in ALSEFV (corresponding to the categories “probable” or “very probable”).

As controls, we selected 25 consecutive patients who completed the treatment with vancomycin without any sign of skin adverse reaction.

### LTT assay

Lymphocyte transformation tests (LTT) were performed following standard procedures in order to confirm the culprit drug (Pichler and Tilch, 2004; Cabañas et al., 2018). The test was performed after the resolution of the clinical symptoms and at least 4 weeks after the end of steroid treatment. Briefly, peripheral blood mononuclear cells (PBMCs) were isolated from anti-coagulated whole blood, and triplicate cultures were established for six days in RPMI culture medium plus 5% autologous serum, in the presence or absence of increasing concentrations (10–200 μg/ml) of vancomycin. ^3^H-thymidine (0.5 μCi/well) was added to the cultures 18 h before harvesting. Proliferation was estimated as ^3^H-thymidine uptake measured in counts per minute (cpm), incorporated into DNA as assessed by liquid scintillation in a β counter (MicroBeta TriLux, Wallac, and PerkinElmer). A stimulation index (SI) was calculated as the ratio of mean cpm values between drug-stimulated and unstimulated cell cultures.

### HLA typing

DNA samples were analyzed by HLA-A*32:01 allele-specific PCR (AS-PCR)/melting curve following the previously published protocol and primers (Rwandamuriye et al., 2019) with minor modifications. Internal control primers were used to amplify the housekeeping gene galactosylceramide (GALC) as described. Primers are shown in Supplementary Table S1. Briefly, the real-time PCR reaction contained 2 μl (100 ng) of total DNA, 1x SYBR Green Master mix (Quantimix Easy kit, Biotools), 250 nmol/L of each HLA-A*32 specific primer, and 50 nmol/L of each GALC primer in a 15 μl final volume. The PCR was performed in 96-well optical plates on a BioRad CFX96 qPCR machine (BioRad Laboratories), and results were analyzed using CFX Manager software (BioRad). In some experiments, amplification products were also visualized on a 1.5% agarose gel.

Preliminary experiments were performed in DNA samples that had previously undergone high-resolution, full allelic HLA typing in the settings of previous studies (Ramírez et al., 2017; Balas et al., 2020) with confirmation that HLA-A*32:01 was amplified only in those cases previously identified as carriers of the allele.

### Statistical analysis

The quantitative data are described as mean and standard deviation, or median, interquartile range (IQR), minimum, and maximum. The qualitative data are described as frequency and percentage.

The nonparametric Mann–Whitney *U* test was applied to compare continuous variables. Fisher’s exact test was used to compare the results of the LTT (positive/negative) in cases with the results in tolerant control donors. Sensitivity and specificity, positive predictive value (PPV), and negative predictive value (NPV) of LTT were calculated using 2 × 2 contingency tables. Receiver operating characteristic (ROC) curves were plotted using a nonparametric method to assess the diagnostic capacity of LTT to vancomycin.

Allele and population frequencies of HLA-A*32:01 were calculated. The association of DRESS with vancomycin exposure was assessed by calculating the odds ratio (OR) and its 95% confidence interval (CI). Fisher’s exact test was used to assess the statistical significance of the differences found between the proportion of individuals carrying the HLA allele among cases and vancomycin-tolerant controls. A *p*-value of <0.05 (two-tailed) was considered statistically significant. Sensitivity (Se), specificity (Sp), positive predictive value (PPV), and negative predictive value (NPV) were computed using 2 × 2 contingency tables. We estimated that the sample size of the study would provide a power ≥80% to detect an OR>5 with a type I error of 0.05.

Statistical analyses were performed using GraphPad Prism v 9.0 (La Jolla, CA, United States).

### Ethical approval

The Research Ethics Committee of University Hospital “Príncipe de Asturias” granted approval for the whole PIELenREd registry and biological sample collection (code PER-MED-2010-01, date: 28 July 2010), under which the present study was carried out. All patients, cases, and controls alike, or their legal representatives provided specific written informed consent for the collection of both personal data and biological samples.

## Results

### Characteristics of patients and controls

Fourteen patients with DRESS associated with vancomycin exposure were included (four possible, seven probable, and three definite DRESS). Drug causality assessment using the ALSEFV identified vancomycin as the possible culprit (score 4–5) in nine cases, probable in two cases (score 6–7), and definite in two cases (score >8). We also included an additional case (P 3) in which ALSEFV scored low on the causal relationship with vancomycin (score = 2), but presented an intradermal test with a positive result in delay reading, thus confirming vancomycin sensitization. Ten (71.4%) were adults and four (28.6%) were children, with an overall median age of 38.5 years (range 3–88), while the median age of the adult population was 66.5 years (range 32–88); nine (64.3%) were women (Table 1). Twenty-five patients who completed the vancomycin treatment (mean exposure time: 14.4 ± 10.2 days) without any sign of skin adverse reaction were included as vancomycin-tolerant controls, with a median age of 64 years (range 28–78), and nine (36%) of them were women (Supplementary Table S2).

**TABLE 1:** Demographics and main results in DRESS cases.

Case	Sex	Age	Ethnic origin	DRESS RegiSCAR score	DRESS RegiSCAR diagnostic	ALSEFV score of vancomycin causality	Time from reaction to LTT	Maximum SI in LTT	HLA-A*32:01 AS-PCR
P_1	Male	83	European	5	Probable	8	NA	NA	Negative
P_2	Female	73	European	7	Definite	4	14 months	31.44	Negative
P_3	Female	39	Mixed	4	Probable	2^a^	8 months	15.13	Negative
P_4	Male	88	European	3	Possible	4	33 days	5.54	Negative
P_5	Female	38	Mixed	3	Possible	4	49 days	9.71	Negative
P_6	Female	58	European	7	Definite	4	38 days^b^	11.97	Positive
P_7	Male	74	European	3	Possible	9	3 months	2.03	Negative
P_8	Female	30	European	4	Probable	5	NA	NA	Negative
P_9	Female	3	European	5	Probable	7	5 months	9.67	Positive
P_10	Male	32	European	6	Definite	4	3 months	3.94	Negative
P_11	Female	12	European	5	Probable	5	3 months	73.03	Positive
P_12	Female	6	European	4	Probable	5	19 months	3.25	Negative
P_13	Female	75	European	4	Probable	6	22 months	38.71	Positive
P_14	Male	6	European	2	Possible	5	84 days	9.87	Positive

NA, not available; AS-PCR, allele-specific PCR.

^a^ Positive delayed intradermal test to vancomycin.

^b^ Similar results were obtained two years and 10 years post-reaction.

### LTT as a diagnostic tool for vancomycin-induced DRESS

**FIGURE 1:**
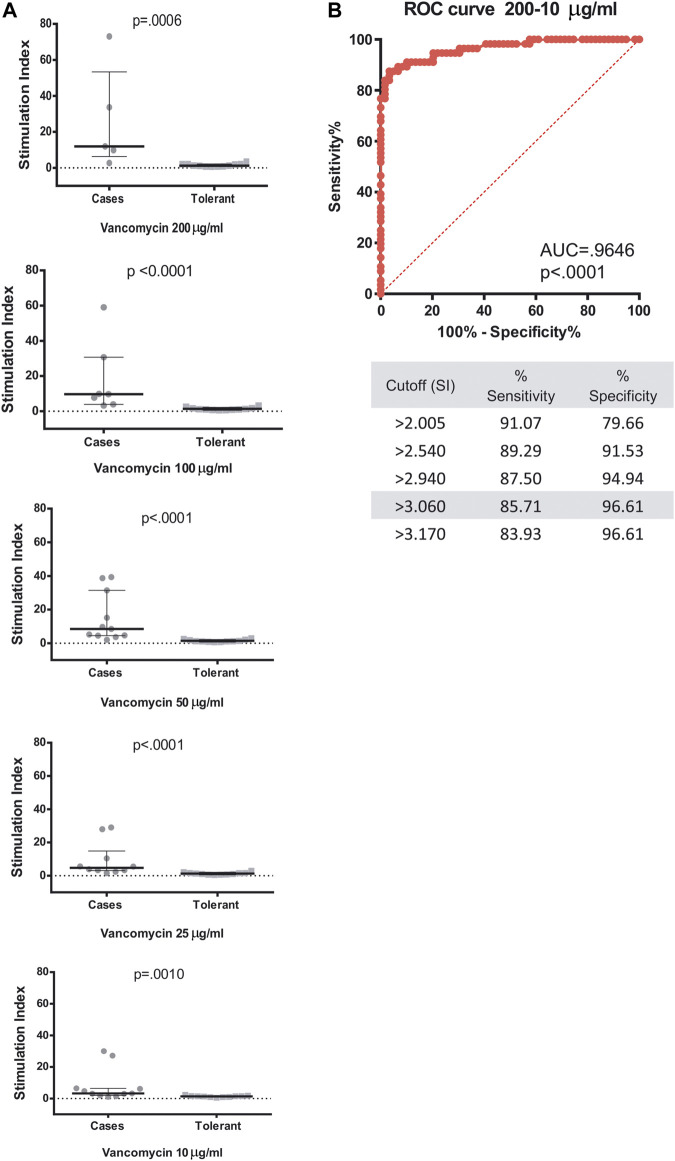
LTT results and ROC curve analysis of LTT to vancomycin. **(A)** PBMCs from 12 patients with a diagnostic of DRESS and vancomycin involvement or from 12 vancomycin-tolerant control donors were isolated and cultured *in vitro* with 10, 25, 50, 100, or 200 μg/ml of vancomycin. Stimulation indices were calculated as described in the methods section. Median and interquartile ranges are shown. Mann–Whitney *U* test was applied for statistical analysis. **(B)** ROC curve analysis, sensitivity, and specificity of grouped results for the five concentrations tested.

Out of 14 cases, we were able to perform the proliferation assays in 12 patients with DRESS attributable to vancomycin (11 with ALSEFV score ≥4 and one with a positive intradermal test). In the remaining two cases, no blood samples during the recovery phase were available (Table 1). LTT was performed in 12 vancomycin-tolerant controls from whom fresh blood samples could be obtained as well. Vancomycin-induced proliferation was tested in a range of five concentrations (10, 25, 50, 100, and 200 μg/ml). Statistically significant differences between cases and controls were found in the proliferative response of lymphocytes at all concentrations (Figure 1A). ROC curves showed a good performance in all concentrations (Supplementary Figure S1). Overall, when results at all concentrations were analyzed together, LTT reached a sensitivity of 85.71% and a specificity of 96.61% when a stimulation index (SI) of >3.06 was considered for positivity (Figure 1B).

Contingency table analysis of LTT results from cases and controls revealed 90.9% sensitivity and 91.67 specificity when the cutoff point for positivity of SI was set at ≥ 3.0, with 90.9 % PPV and 91.67% negative predictive value (NPV). On the other hand, when the SI cutoff point was set at ≥ 2.0, sensitivity and NPV increased to 100%, but specificity decreased to 58.33% and PPV was 68.75% (Table 2 and Supplementary Table S3).

**TABLE 2:** Summary of sensitivity and specificity of LTT to vancomycin during the recovery phase of DRESS patients according to different cutoff points considered for positivity.

Cutoff point	Sensitivity (%)	Specificity (%)	PPV (%)	NPV (%)	Fisher’s exact test
SI ≥ 3	90.9	91.67	90.9	91.67	*p* = 0.0001
SI ≥ 2	100	58.33	68.75	100	*p* = 0.0046

### HLA associations with vancomycin-induced DRESS

To validate the AS-PCR HLA-A*32:01 typing assay (Rwandamuriye et al., 2019) in our laboratory, DNA from 43 donors previously genotyped for HLA-I alleles with high resolution in previous studies involving SCARs to anticonvulsants (Ramírez et al., 2017) or benznidazole (Balas et al., 2020) was tested using real-time PCR as described in the Methods section. Samples were identified as positive or negative based on the presence or absence of HLA-A*32:01-specific melt peaks and confirmed in agarose electrophoresis (Supplementary Figure S2). Only three samples from patients previously identified as carriers of the HLA-A*32:01 allele were accurately identified as positive, and all the remaining 40 donors were negative. HLA alleles previously identified in negative samples are listed in Supplementary Table S4. AS-PCR assays were subsequently performed in all 14 DRESS cases associated with vancomycin, to check for the presence of the risk HLA-A*32:01 allele. Five cases (35.7%) were identified as carriers. Interestingly, 75% (3/4) of pediatric cases were carriers of the risk allele, while it was identified in only 20% of adults (40% of adults if only probable or definite DRESS cases with positive LTTs are included). As a general population group for comparison, we considered a published group of 253 hematological Spanish donors with a population frequency of 9.5% carriers of the allele (Balas et al., 2011). The difference between the DRESS group and the general population group was statistically significant (*p* = 0.011; Fisher’s exact test), representing an OR 5.30 (95% CI: 1.64–17.10).

The odds ratio was slightly higher (OR 6.36) when only DRESS cases with a probable or definite diagnosis (DRESS score ≥4) were considered in the analysis. When we restricted the analysis to the subset of eight cases with a DRESS score ≥4 and a SI ≥ 3 in LTT, the frequency of HLA-A*32:01 carriers rose to 50% and the OR increased to 9.54 as compared to the general population (*p* = 0.0056).

The presence of the risk HLA-A*32:01 allele was also investigated by AS-PCR assay in 25 vancomycin-tolerant control donors, being identified in only one of them (4%). When compared to such a tolerant group, the association between the risk allele and vancomycin-induced DRESS yielded an OR of 13.33 (95% CI: 1.36–130.30; *p* = 0.016), and was stronger when only cases with positive LTT (SI ≥ 3) were considered (OR 24.0; 95% CI: 2.28–252.60; *p* = 0.0040). Similar results were found when only probable or definite DRESS cases were included in the analysis (OR 24.0; 95% CI: 2.10–273.86; *p* = 0.0076) (Table 3).

**TABLE 3:** HLA-A*32:01 frequency in vancomycin-induced DRESS cases as compared to population controls and vancomycin-tolerant controls.

Population control				
	HLA-A*32:01 allele frequency		OR (95% CI)	*p*-value^b^
	DRESS	General population^a^		
DRESS score ≥2	5/14 (35.7%)	24/253 (9.5%)	5.301 (1.643–17.102)	0.011
DRESS score ≥4	4/10 (40%)	24/253 (9.5%)	6.361 (1.677–24.129)	0.014
LTT vancomycin SI > 3				
DRESS Score ≥2	5/11 (45.45%)	24/253 (9.5%)	7.95 (2.257–28.022)	0.0033
DRESS score ≥4	4/8 (50%)	24/253 (9.5%)	9.54 (2.241–40.62)	0.0056

**Vancomycin-tolerant control**				
	HLA-A*32:01 allele frequency		**OR (95% CI)**	***p*-value** **^b^**
	DRESS	**Vancomycin tolerant**		
DRESS score ≥2	5/14 (35.7%)	1/25 (4.%)	13.33 (1.36–130.30)	0.016
DRESS score ≥4	4/10 (40%)	1/25 (4%)	16.0 (1.50–170.62)	0.017
LTT vancomycin SI > 3				
DRESS score ≥2	5/11 (45.45%)	1/25 (4%)	24.0 (2.28–252.60)	0.0040
DRESS score ≥4	4/8 (50%)	1/25 (4%)	24.0 (2.10–273.86)	0.0076

^a^ From Balas et al. tissue antigens (2011).

^b^ Fisher’s exact test.

## Discussion

The main findings of the present case-control study are the following: 1) LTT as a diagnostic tool to identify vancomycin sensitization showed high sensitivity and specificity, as well as positive predictive and negative predictive values (all of them over 90%) when the cutoff point of SI for positivity was set at ≥ 3, using the ALSEFV drug causality algorithm as the gold standard; 2) the presence of HLA-A*32:01 allele was strongly associated with validated DRESS cases in whom vancomycin was suspected to be the culprit drug, when compared to both population controls and vancomycin-tolerant controls, confirming it as a relevant biomarker of susceptibility in a European-Spanish population.

SCARs are T-cell-mediated type IV hypersensitivity reactions that cannot be predicted based on the pharmacological characteristics of the drug alone, and are responsible for significant morbidity, mortality, and socioeconomic costs (Gomes and Demoly, 2005). The key to prevention from further exposure to the culprit drugs involves the correct identification of the causative drug through a combination of *in vitro* and/or *in vivo* tests (Alfirevic and Pirmohamed, 2017; Ardern-Jones and Mockenhaupt, 2019; Pirmohamed, 2019) and, therefore, allow patients to receive treatments that otherwise might not have been permitted in the future if the patient is labeled as being allergic. Rechallenge *in vivo* tests are contraindicated in DRESS cases. On the other hand, cutaneous tests have high specificity but low sensitivity. In this scenario, *in vitro* tests are recommended as a first approach to determine the culprit drugs (Mayorga et al., 2016). In a previous study, we found that *in vitro* LTT tests have good specificity and sensitivity in DRESS cases when performed upon resolution of the clinical symptoms (Cabañas et al., 2018). Nonetheless, vancomycin-specific LTT showed lower specificity, and previous reports had suggested non-specific induction of lymphocyte proliferation by vancomycin (Pichler and Tilch, 2004). In the present study, the ROC curve analysis in cases and vancomycin-tolerant controls confirmed the suitability of the LTT as a tool to evaluate vancomycin sensitization in DRESS cases, with good sensitivity, specificity, PPV, and NPV when a SI ≥ 3 is considered as the cutoff point. In our previous study, tolerant donors were not analyzed, and we used ALSEFV scores as standard and SI ≥ 2 as criteria for positivity, which results in lower specificity and PPV.

No specific algorithm has been developed for drug causality assessment in DRESS. The Naranjo score (Naranjo et al., 1981) has been classically used to evaluate adverse drug reactions. We have used the algorithm of the Spanish pharmacovigilance system (ALSEFV) as recommended by the Spanish guidelines for the management of DRESS (Cabañas et al., 2020). There are no data available on the specificity of the ALSEFV to accurately determine the culprit drugs; however, a previous study suggested a good agreement with rechallenge results in a variety of non-immediate drug reactions including mild reactions (Cabañas et al., 2018). Nonetheless, not all mild delayed reactions are necessarily T-cell mediated. Our LTT results, after comparison of cases with tolerant donors, suggest that LTT is a sensitive and specific tool to identify individuals with DRESS reactions to vancomycin when performed after resolution of the clinical symptoms.

Genetic testing has a potential role among strategies used for prevention in identifying whether an individual may be susceptible to developing a serious adverse reaction from a particular drug, as pharmacogenomic studies have revealed strong associations between SCARs and genes encoding HLA molecules in a drug and ethnicity-specific pattern (Su et al., 2016; Phillips, 2018; Kuruvilla et al., 2022). Thus, pharmacogenetic testing in SCARs has been proposed for prevention, monitoring, and diagnosis (Pirmohamed, 2019). We used the recently published HLA-A*32:01 AS-PCR assay (Rwandamuriye et al., 2019) to confirm the feasibility of this specific test for identifying carriers of the risk allele and to evaluate its association with vancomycin-induced DRESS in a group of Spanish-European patients. Our study confirms that HLA-A*32:01 AS-PCR is a reliable assay, as well as the previously described association, although with lower OR than that in US citizens of European descent (OR 70 in US cases vs*.* 24 in Spanish cases). The strongest associations were found when cases were restricted to those showing positive LTT results (SI > 3). However, even when only the more strict criteria (only probable or definite DRESS cases with LTT SI > 3) were used for analysis, only 50% of Spanish cases were carriers of the allele as compared to 82.6% in the American group. Interestingly, we observed a higher proportion of carriers of the risk HLA-A*32:01 allele among children with vancomycin-induced DRESS. Given that only four children were included in the analysis, further studies including larger cohorts would be needed to draw specific conclusions in pediatric cases and to explore the underlying mechanisms in case of confirmation of this finding. On the other hand, as LTT specificity is not 100%, we cannot rule out the possibility of including a false-positive adult patient in the analysis that, due to the small sample size, could skew the results. Nonetheless, the discrepant strength of the association in the whole population may also be related to a dissimilar frequency of other HLA alleles that might also be involved in the presentation of vancomycin to specific TCRs. Glycopeptide antibiotics contain a heptapeptide core structure, and molecular docking analysis predicted the binding of vancomycin within the peptide-binding groove of HLA molecules in the absence of other peptides (Konvinse et al., 2019). Moreover, vancomycin, as well as other glycopeptide antibiotics such as teicoplanin and telavancin, were also predicted to bind HLA-DQ (DQA1*01:01, DQB1*05:03) as the molecular basis for cross-reactive T-cell responses (Nakkam et al., 2020). It is thus possible that additional HLA class I or class II alleles present in our population might be responsible for vancomycin-specific DRESS, and this issue deserves further research. In this sense, only one of our cases (P14) was tested for teicoplanin with a maximum SI = 7.0, strongly suggesting cross-reactivity with teicoplanin. The patient was a carrier of HLA-A*32:01. However, no information is available regarding other HLA class I or HLA class II alleles, and to speculate about the possible cross-reactivity among glycopeptide antibiotics in our cases would be too risky. Among the limitations of the study, we should mention the following: first, the number of cases analyzed was small, though it proved enough to detect statistically significant strong associations; second, we used the algorithm of the Spanish pharmacovigilance system (ALSEFV) as the gold standard to identify vancomycin as the culprit drug which is a tool far from perfect, and thus, the parameters estimated for LTT and HLA-A*32:01 performance can only be considered as approximate estimates in the absence of a better gold standard; third, the sample of vancomycin-tolerant controls were selected in a consecutive manner, but not at random, and it may not be representative enough of the whole population exposed to vancomycin; however, it is important to note that the HLA-A*32:01 allele frequency among population controls was fairly similar, which reinforces the validity of the results obtained.

In conclusion, our study confirms HLA-A*32:01 association with vancomycin-induced DRESS in an independent group of European cases, and suggests that the combination of HLA-A screening for this allele as well as *in vitro* LTT test could be useful to identify DRESS patients sensitized to vancomycin. Although a negative AS-PCR test does not exclude vancomycin sensitization, a positive test could be helpful to identify cases before LTT can be performed. Moreover, PCR is a technique widely available in clinical laboratories. Furthermore, research is needed to confirm these findings in other European populations. Finally, it is important to stress that the usefulness of testing the alleles to prevent vancomycin-induced type IV hypersensitivity reactions should be specifically examined in prospective studies.

## Data Availability

The original contributions presented in the study are included in the article/Supplementary Material; further inquiries can be directed to the corresponding author.
