# Supplementary material for: LTT and HLA testing as diagnostic tools in Spanish vancomycin-induced DRESS cases: A case-control study
Source: Front Pharmacol. 2022 Oct 20;13:959321. doi: 10.3389/fphar.2022.959321 (PMC9631441; doi:10.3389/fphar.2022.959321)
Supplement: Supplementary file 1 [file DataSheet1.pdf]

Supplementary Material

1 Supplementary Tables

Supplemental Table S1 Primer Sequences for HLA Typing of HLA-A*32:01 Allele

Primer Description	Sequence	Target<br><br>Predicted product length (bp)	Tm peaks
HLA-A*32 forward primer	5'-GACGACACGCAGTTCGTGCGGTT+T-3'	HLA-A*32<br><br>157 bp	88.5ºC
HLA-A*32 reverse primer	5'-GAGCGCGATCCGCAGGC-3'		
GALC forward primer	5'-TTACCCAGAGCCCTATCGTTCT-3'	GALC<br><br>352 bp	76ºC
GALC reverse primer	5'-GTCTGCCCATCACCACCTATT-3'		

Plus sign marks the location of a locked nucleic acid in the primer sequence.
Tm: Melting temperature

Supplemental Table S2. Vancomycin tolerant control donors

Code	Sex	Age	LTT Vancomycin	HLA-A32:01
Vanco_tol_1	Male	64	ND	NEG
Vanco_tol_2	Male	47	ND	NEG
Vanco_tol_3	Female	48	ND	NEG
Vanco_tol_4	Female	64	ND	NEG
Vanco_tol_5	Female	28	ND	NEG
Vanco_tol_6	Male	64	NEG	NEG
Vanco_tol_7	Male	60	SI=2.14	NEG
Vanco_tol_8	Male	64	NEG	NEG
Vanco_tol_9	Male	74	SI=2.08	NEG
Vanco_tol_10	Female	64	NEG	NEG
Vanco_tol_11	Male	71	SI=2.53	NEG
Vanco_tol_12	Female	66	NEG	NEG
Vanco_tol_13	Female	55	NEG	NEG
Vanco_tol_14	Male	78	SI=3.48	NEG
Vanco_tol_15	Male	75	NE	POS
Vanco_tol_16	Female	68	ND	NEG
Vanco_tol_17	Male	50	ND	NEG
Vanco_tol_18	Male	73	ND	NEG
Vanco_tol_19	Male	64	NEG	NEG
Vanco_tol_20	Male	73	SI=2.65	NEG
Vanco_tol_21	Male	65	NEG	NEG
Vanco_tol_22	Female	61	ND	NEG
Vanco_tol_23	Male	56	ND	NEG
Vanco_tol_24	Female	53	ND	NEG
Vanco_tol_25	Male	67	ND	NEG

ND: Not done; NE: not evaluable; NEG: Negative; POS: Positive; SI: Stimulation index (the maximum value of SI is shown for those cases with SI≥2)

Supplemental Table S3. Contingency tables. Analysis of the performance of LTT in Vancomycin-induced DRESS cases

Analysis considering a Stimulation index (SI) ≥ 3 as positive result				
	Cases	Tolerant controls	Sum	Predictive value
LTT positive (SI≥3)	10	1	11	PPV= 90.9%
LTT negative (SI<3)	1	11	12	NPV=91.67%
Sum	11	12	**23**	
Sensitivity (Se) and Specifity (Sp)	Se= 90.9%	Sp= 91.67%		p= 0.0001<br>Fisher's exact test
**Analysis considering a Stimulation index (SI) ≥ 2 as positive result**				
	Cases	Tolerant controls	Sum	Predictive value
LTT positive (SI≥2)	11	5	16	PPV= 68.75%
LTT negative (SI<2)	0	7	7	NPV=100%
Sum	11	12	**23**	
Sensitivity (Se) and Specifity (Sp)	Se= 100%	Sp= 58.33%		p=0.0046<br>Fisher's exact test

Supplemental Table S4. HLA-A alleles in 40 individuals that tested negative in the HLA-A*32:01 AS/PCR assay

HLA-A specificities	n
A*0101	1
A*0201	22
A*0205	3
A*0222	2
A*0301	5
A*0302	1
A*1101	1
A*2301	2
A*2402	8
A*2501	1
A*2601	3
A*2902	1
A*3002	2
A*3101	6
A*3301	1
A*3303	1
A*6801	5
A*6802	3
A*6817	8
A*8001	1

Supplemental Table S5. Other potentially implicated drugs

P_1	Piperacillin/Tazobactam
P_2	Cefepime
P_3	Piperacillin/Tazobactam, Ceftriaxone, Meropenem
P_4	Meropenem
P_5	Clarithromycin, Amoxicillin/Clavulanic, Meropenem, Levofloxacin, Metamizol
P_6	Cefepime, Meropenem
P_7	None
P_8	Metamizol
P_9	Meropenem, Acetazolamide
P_10	Cloxacillin, Pantoprazol
P_11	Cefotaxime
P_12	Ceftriaxone
P_13	Ceftazidime
P_14	Levofloxacin

2 Supplementary Figures

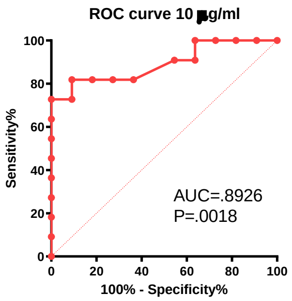

Cutoff (SI)	%Sensitivity	%Specificity
> 1.800	81,82	90.91
> 2.160	72,73	90.91
> 2.670	72,73	100,0

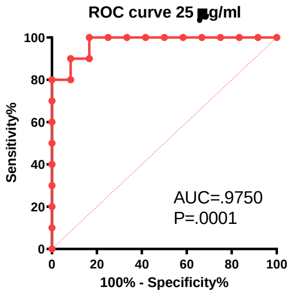

Cutoff (SI)	%Sensitivity	%Specificity
> 1.800	90.00	83.33
> 2.160	90.00	91.67
> 2.670	80.00	91.67

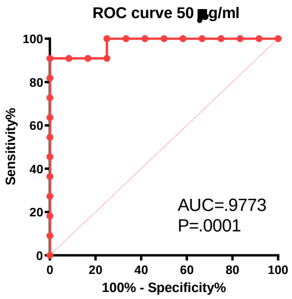

Cutoff (SI)	%Sensitivity	%Specificity
> 2.730	90.91	91.67
> 3.320	90.91	100.0
> 4.110	81.82	100,0

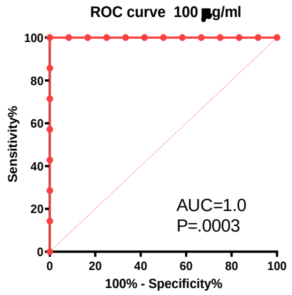

Cutoff (SI)	%Sensitivity	%Specificity
> 2.130	100.00	83.33
>2.885	100.00	91.67
>3.235	100.00	100,0
>3.590	85.71	100,0

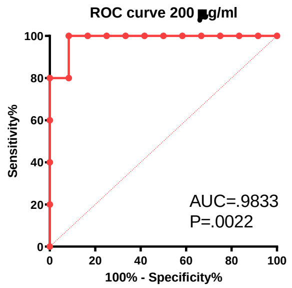

Cutoff (SI)	%Sensitivity	%Specificity
>2.115	100.00	83.33
>2.430	100.00	91.67
>3.100	80.00	91.67

Supplementary Figure S1. ROC curve analysis of LLT results to vancomycin at each one of the concentrations tested
